# Evidence-based medicine, shared decision making and the hidden curriculum: a qualitative content analysis

**DOI:** 10.1007/s40037-020-00578-0

**Published:** 2020-04-22

**Authors:** Emélie Braschi, Dawn Stacey, France Légaré, Roland Grad, Douglas Archibald

**Affiliations:** 1 Lamont Primary Health Care Research Centre, Ottawa, ON Canada; 2 Faculty of Health Sciences, Ottawa Hospital Research Institute, Ontario, ON Canada; 3 Department of Family Medicine and Emergency Medicine, Faculty of Medicine, Laval University, Québec, QC Canada; 4 Herzl Family Practice Centre, Montreal, Canada

**Keywords:** Evidence-based medicine, Shared decision making, Hidden curriculum

## Abstract

**Introduction:**

Medical education should portray evidence-based medicine (EBM) and shared decision making (SDM) as central to patient care. However, misconceptions regarding EBM and SDM are common in clinical practice, and these biases might unintentionally be transmitted to medical trainees through a hidden curriculum. The current study explores how assumptions of EBM and SDM can be hidden in formal curriculum material such as PowerPoint slides.

**Methods:**

We conducted a qualitative content analysis using a purposive sample of 18 PowerPoints on the management of upper respiratory tract infections. We identified concepts pertaining to decision making using theory-driven codes taken from the fields of EBM and SDM. We then re-analyzed the coded text using a constructivist latent thematic approach to develop a rich description of conceptualizations of decision making in relation to EBM and SDM frameworks.

**Results:**

PowerPoint slides can relay a hidden curriculum, which can normalize: pathophysiological reasoning, unexplained variations in clinical care, the use of EBM mimics, defensive medicine, an unrealistic portrayal of benefits, and paternalism.

**Discussion:**

Addressing the hidden curriculum in formal curricular material should be explored as a novel strategy to foster a positive attitude towards EBM and SDM and to improve patient outcomes by encouraging the use of these skills.

**Electronic supplementary material:**

The online version of this article (10.1007/s40037-020-00578-0) contains supplementary material, which is available to authorized users.

## Introduction

In the 1970s, quality concerns were raised in the United States as differences in regional rates of hospitalization and surgical intervention were noted [1]. These variations were “associated with differences in beliefs among physicians concerning the indications [of medical treatments]”, and stemmed from a lack of empirical data to compare one treatment against another [1]. These concerns led to the emergence of the fields of evidence-based medicine (EBM) and shared decision making (SDM). EBM is “the conscientious, explicit, and judicious use of current best evidence in making decisions about the care of individual patients” [2]. It addresses how to formulate a question, search the literature and appraise the quality of the evidence to ensure that clinical decision making is based on the best available evidence as opposed to “intuition, unsystematic clinical experience, and pathophysiological rational” [2]. EBM has sometimes been portrayed as a cookbook: evidence from clinical trials dictates clinical decision making [3]. This view has been widely criticized as it fails to acknowledge that population-wide data may not apply to individual patients with unique clinical and social contexts [4]. In addition, obtaining the “best-available evidence” has been challenging as clinical trials have many limitations. For example, patients with comorbidities and frailty are often excluded, and results may be biased by poor methodology [4]. In fact, most medical decision making includes some level of uncertainty. Each option comes with potential benefits and harms. SDM addresses these uncertainties by emphasizing patients’ key role as a partner in decision making. For example, one framework advocates the use of a “choice talk” that ensures patients are aware of the different options available, followed by an “option talk” that describes the harms and benefits of each option using the best available evidence [5]. Finally, a “decision talk” focuses on eliciting patients’ preferences and moving towards a decision [6].

Educational frameworks such as CanMeds include EBM and SDM competencies [7]. In North America and Europe, these objectives have traditionally been fulfilled with specific EBM and SDM training programs [8, 9], but it is not clear whether these lead to the long-term mastery of those concepts [10]. Misconceptions have been reported: some medical trainees feel uneasy with giving patients the option of not taking a medication or of not undergoing a medical procedure from which they may benefit (a central aspect of SDM) [11]. In fact, clinicians have a tendency to overestimate benefits of interventions and underestimate harms [12]. Clinicians also overestimate their EBM knowledge [13] and sometimes believe that patients do not want SDM [14, 15], even though patients and families often want to be engaged in their care [16–18].

Given that clinicians play an important role in training medical trainees, it is possible that these misconceptions of EBM and SDM have inadvertently been transmitted to medical students through a hidden curriculum [19]. The hidden curriculum describes the “understandings’, customs, rituals, and taken for granted aspect of what goes on in the life space we call medical education” [20]. It addresses unintended messages that may be transmitted during medical training and that may or may not align with official educational objectives [20]. For example, a hidden curriculum that inadvertently portrays clinical medicine as opinion-based and paternalistic might have a negative influence on how medical trainees perceive EBM and SDM. This might in turn negatively affect patient care. Ideally, during medical training, clinical decision making should model a systematic approach to the gathering and synthesizing of the evidence (the focus of EBM). The best available evidence should then be applied to the specific clinical context of the patient, benefits and harms communicated, and values clarified to tease out the importance, for that particular patient, of benefiting from an intervention versus avoiding harms (the focus of SDM) [21].

A hidden curriculum may be present in all aspects of medical education [20] but formal teaching environments represent a valuable opportunity: they are ubiquitous throughout medical training and curriculum material can be monitored. In order to be addressed, however, a hidden curriculum first needs to be exposed [22], and it is not known to what extent curricular material can relay hidden messages that may conflict with the official curriculum’s learning objectives. The present study was therefore undertaken to explore how assumptions of EBM and SDM can be hidden in formal curricular material.

## Methods

We followed the Standards for Reporting Qualitative Research to guide the writing of this study [23]. No ethical review was required for this work as it relied on publically available documents. In addition, in accordance to the Declaration of Helsinki, identifiable information was removed.

### Data collection

We were interested in exploring formal curriculum documents. Given the ubiquitous nature of PowerPoint slides to deliver clinical content and their public accessibility on the internet, we decided to use a purposive sample of PowerPoint slides. An SDM training program that used PowerPoint slides on the diagnosis and management of upper respiratory tract infections (URTIs) has been shown to decrease the use of antibiotics (DECISION + workshop) [18]. We therefore decided to focus on this topic, in particular on the management of sinusitis, pharyngitis and bronchitis. We conducted Google searches on 14 October 2017 with the following terms: “medicine slides pharyngitis”, “medicine slides acute sinusitis”, and “medicine slides bronchitis”. Both the “All” and “Images” options were selected. We only downloaded or copied PowerPoint slides that were authored by a medical professional, as determined by an affiliation with a medical school, a medical department or by an official recognition such as Dr., MD, or Professor. All of the PowerPoints identified using this method [16] were included in the analysis. After reviewing the slides, we realized that only a few contained EBM and SDM content. To enrich our sample we also searched MedEdPortal (www.mededportal.org), a repository of open access, peer-reviewed teaching materials with the following terms: “pharyngitis”, “sinusitis”, and “bronchitis”. Finally, we used the workshop slides on the management of URTI for primary care physicians developed by a leader in the field of SDM (DECISION + workshop) [18]. We read each PowerPoint and transcribed sections describing the diagnosis and management of URTI into an Excel spreadsheet. We described images and figures, when appropriate. We also transcribed memos recording first impressions and reactions to the PowerPoint slides during the data collection process. We then imported the Excel spreadsheet into the NVivo qualitative data analysis software.

### Data analysis

We selected two frameworks to guide the analysis. From the field of EBM, we used the Sicily statement on evidence-based practice. It is a consensus statement from an international working group that outlines the skills required to practice according to evidence-based principles [24]. These include the formulation of answerable questions to address knowledge gaps and of effective search and appraisal strategies. In addition, students should be able to critically appraise evidence for validity and to reflect on its clinical importance and on its value within the patient’s specific context [24].

From the field of SDM, we chose the International Patient Decision Aid Standards (IPDAS) collaboration criteria [25]. These criteria focus on whether key elements are presented to the reader in sufficient detail to enable SDM [26, 27]. These elements include a description of the health condition and of its natural course, an explicit statement indicating the decision to be considered and a list the possible options. For each option, positive and negative features should be described so that they can be compared (for example, by quantifying negative and positive features using event rates). Statements to help patients navigate their preferences in relation to the different options should be provided. Finally the development process of the decision aid, knowledge synthesis strategies and funding should be made explicit [26]. These criteria have been used for the evaluation of continuing medical education articles [27–29].

We first conducted a directed qualitative content analysis [30]. We identified material that conceptualized concepts relevant to EBM and SDM with codes generated from the IPDAS criteria and the Sicily EBM statement. We then re-analyzed the coded material to expose the presence of a hidden curriculum. We chose a latent thematic approach to develop themes that captured concepts beyond the surface meaning of the data [31]. The research paradigm was constructivist and the perspective of each of the authors was considered an enriching factor [32]. EB is a recent graduate of the Family Medicine residency with an interest in EBM and SDM. DS and FL are pioneers in the field of SDM and have researched SDM training programs. RG is an academic family physician who has published in the fields of EBM and SDM. DA is a medical education researcher with an interest in reflective learning. To extract latent, hidden, assumptions regarding SDM and EBM, EB used an inductive, iterative process, whereby each of the different PowerPoints were compared among themselves and the chosen frameworks. EB reflected on the material from the perspective of a recent medical trainee and of a scholar with in depth knowledge of EBM and SDM to extract latent, hidden, assumptions regarding SDM and EBM. Coding was informed by ongoing discussions with DS and DA to maintain reflexivity. Checks with an external expert (RG) supported the validity of these analyses.

## Results

**Fig. 1:**
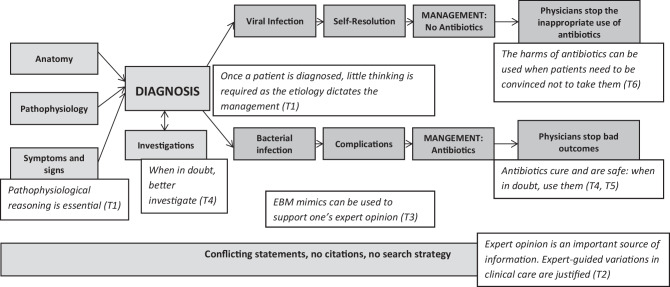
Concept mapping of a PowerPoint on the management of URTI that is at odds with EBM and SDM frameworks. Implicit messages are shown with the corresponding themes. *T1*: Pathophysiological reasoning, *T2*: Unexplained variations in clinical care, *T3*: Use of EBM mimics; *T4*: Defensive medicine, *T5*: Unrealistic portrayal of benefits, and *T6*: Paternalism. Variations of this approach were common. Pathophysiological reasoning links diagnosis, etiology and clinical management. Viral infections are presented as self-limited and harms of antibiotics can be used to convince patients to avoid taking them. Bacterial infections, however, may lead to complications and antibiotics are needed to “cure” these patients; antibiotics are therefore presented as safe

Of the 18 PowerPoints, 11 covered sinusitis, 9 pharyngitis and 5 bronchitis (Supplemental Table 1). We identified multiple misconceptions of EBM and SDM in these PowerPoint slides (Fig. 1). Although present to various degrees across the slides, the overarching message was that viral and bacterial infections are different entities. On the one hand, viral infections were presented as self-limited infections that should not to be treated with antibiotics (Fig. 1). The harms of antibiotics could be used in such instances to steer patients away from taking antibiotics. On the other hand, bacterial infections were presented as severe, non-resolving, and as putting patients at risk of complications; antibiotics were therefore needed to cure these patients (Fig. 1). These messages were coupled with the predominant use of expert opinion, unreferenced claims, and the inadequate use of EBM.

### Pathophysiological reasoning

Understanding pathophysiological processes to guide clinical management was almost universally endorsed by authors. Whether a condition was felt to be viral or bacterial appeared more important than whether viral infections could be distinguished from bacterial infections in clinical practice or whether antibiotics had been shown effective in these particular clinical contexts. This “diagnostic binning” directed management by drawing a linear relationship between etiology and management:* “Viral infections need only symptomatic treatment. Bacterial infections need antibiotics in addition of symptomatic treatment” (P12). *In other words, clinical reasoning was restricted to “What management matches the presumed etiology?” The problem with this approach is that it makes the wrong assumption that in clinical practice clinicians may easily differentiate between viral and bacterial infections. In contrast, SDM/EBM approaches do not attempt to separate symptoms and signs under viral or bacterial labels. When using EBM to support clinical decision making, making a diagnosis and understanding the pathophysiology is not as important as understanding the inclusion and exclusion criteria of trials evaluating treatment effectiveness. One needs to determine whether patients with similar presentations benefit or not from a specific management option. None of the 18 PowerPoints, however, were able to fully embrace this concept, leading to diagnostic clinical decision rules with different criteria than the ones used to assess benefits from antibiotics *(e.g. for sinusitis: P14, P17, P18). *For example, P14 references a guideline that states that bacterial sinusitis is diagnosed based on symptom duration, purulent nasal secretion and facial tenderness. However, the same author references a meta-analysis showing that neither the duration of illness nor the presence of purulent nasal discharge are good at predicting who will benefit from antibiotics (P14).

### Unexplained variations in clinical care

Some PowerPoint slides made contradictory statements (for example P16 and P17 had mutually exclusive statements regarding the usefulness of mucopurulent discharge for the diagnosis of bacterial sinusitis, neither with a reference). These statements were not supported by any rational, which normalizes expert-guided variations in clinical care, whereby one simply chooses a course of action, with little explanation and no consideration of the best available evidence or of patients’ values and preferences. Statements such as *“Expert opinions vary on the appropriate role of antibiotics in treating acute sinusitis” (P15)* reinforced this message as no explanations for these variations were given. An EBM/SDM approach would consider the validity and limitations of the best available evidence, the particular clinical context, and patients’ values and preferences to explain why physicians may chose a different course of action when faced with similar scenarios.

### Use of EBM mimics

Authors often used “EBM mimics” that gave the false impression that their claims were grounded in EBM principles. Clinical decision tools, such as the Centor score, can be used to guide clinical management by providing the probability of a particular bacterial infection (Group A Streptococcus (GAS)) based on a set of clinical criteria [33]. However, these decision tools were rarely used properly. For one author it morphed into a *“classic triad” (P7)*, which gave the impression that patients presenting with those symptoms “must” have GAS pharyngitis, which is inconsistent with the best available evidence [33]. In other instances prescriptive terminology ignored the nuanced use of pre- and post-test probabilities, which became *“low”, “moderate” *and* “high”* scores *(P12, similar in P13)*. Instead of using a “high” score, which could have a different meaning to different people, a statement that aligns with EBM/SDM approaches would use *“a patient with 3–4 criteria has a 40–60% chance of having GAS” (P13)*. Furthermore, authors used EBM-like terminology, such as “trustworthy” (P6), (does it mean sensitive? specific?) or failed to provide context. For example, “*highest positive predictive value*” (*P16*) was used, but what this refers to was not indicated (does it help predict response to antibiotics?), and the numerical value was missing (it might be the “highest” with little clinical utility). These EBM mimics create the illusion that one is using the best available evidence and normalize the distorted use of EBM to support personal opinion.

### Defensive medicine: Investigations

Authors gave mixed messages regarding the utility of investigations for the diagnosis of URTI, inadvertently suggesting that “more is better” (Fig. 1). For sinusitis, only one author was explicit about investigations being *“not necessary” (P5).* Most only used vague wording when suggesting that investigations were not needed (*“not usually needed” (P7, similar in P1, P6)*). In addition, numerous slides were sometimes allocated to investigations *(P2: 8 slides on x‑ray)* and across all PowerPoints, a menu of investigations was presented (7 in total), watering down the suggestion that these were typically not indicated.

### Defensive medicine: Management

All of the studied URTIs are self-limited conditions, whether viral or bacterial. However, authors inadvertently presented bacterial etiologies as the “real” infections, thereby promoting the over-management of usually benign conditions. This happened when the natural history was made explicit for viral illnesses only (P3, P7, P8) whereas persistent symptoms were linked solely to bacterial illnesses (P1, P3, P5, P7). Authors also implied that untreated bacterial infections would lead to complications (P6, P2, P10). For example: *“serious complications rare when managed properly (P15, similar in P7, P9)” *implies that without antibiotics patients may suffer from complications. Furthermore, “complications” were presented with no incidence rates, unintentionally inflating the risk. The active management of bacterial infection was therefore advocated and poor outcomes were linked to under-management, inadvertently relaying the message that overtreatment is the safest option. In fact, URTIs are self-resolving illnesses; in ~80% of patients with pharyngitis (whether viral or bacterial) the symptoms will resolve within 7 days [34]. Rates of complications are so low that they do not typically justify the use of antibiotics [34, 35].

### Unrealistic portrayal of benefits

There was an almost-universal lopsided presentation of the benefits of antibiotics, which gave the impression that antibiotics cure all patients. Benefits were usually not quantified and included decreased symptom burden *(P2, P8, P10, P12)*, decreased complications *(P8, P9, P10, P12)*, decreased recurrence *(P2)* and decreased transmission *(P8, P10, P12)*. Wording such as “*eradicate*” (*P6*) or “*control*” (*P2*) suggested a resounding effectiveness of antibiotics. A minority of authors attempted to quantify benefits using different methods; however, formats such as event rates in the control and antibiotic group, recommended for SDM [27], were uncommon *(P14, P18). *Furthermore, benefits were sometimes quantified only in the setting of antibiotics, with no placebo response provided, misleading the learner into thinking that antibiotics are exceedingly effective (*P16, P17*). In fact, URTIs being self-resolving illnesses, the majority of patients do not benefit from the use of antibiotics with a number needed to treat of ~5–20 for resolution of symptoms [34–36].

### Paternalism

Physicians were typically presented as the ones responsible for making the decision of initiating antibiotics or not, and for convincing patients to follow their plans. For example: *“How should clinicians decide whether to use antibiotics to treat acute sinusitis?” (P15)* designates the physician as the decision maker with no input from the patient. In fact, there was a relative absence of the word “patient” (patients are mentioned only in *P12, P14, P17, P18*), and the tone of some statements was overtly paternalistic *(“Patients do not need antibiotics to feel satisfied”) (P12).*

The number needed to harm for adverse events with the use of antibiotics is ~10–25 [35, 36]. Instead of presenting the harms of antibiotics in an unbiased way, when the recommendation was for patients to take antibiotics, harms were not mentioned or were minimized. Only one author mentioned a side effect in the context of a bacterial illness (P17). However, when the authors’ recommendation was for patients not to take antibiotics then the authors more consistently stated the harms of antibiotics *(“promotes antibiotic resistance, adverse reactions such as allergy and anaphylaxis, costly”) (P12, similar in P8, P14, P16)*. One author even suggested that harms could be used to persuade patients not to use antibiotics (P12).

## Discussion

Our results expose misconceptions of EBM and SDM hidden in our sample of PowerPoint slides. Educators that design formal curricula to foster EBM and SDM skills need to address all curricular elements that may impact how students value these skills [37]. Our results suggest that PowerPoint slides may contribute to the hidden curriculum and that “day-to-day” standard didactic lectures may reflect an underlying microculture at odds with EBM and SDM frameworks. Students may be taught the importance of involving patients in clinical decision making during specific SDM curricula. However, if in all of their other clinical didactic lectures decision making appears primarily paternalistic, then the conflicting messages will marginalize the importance of using SDM for patient care, and will misrepresent the expectations of medical practice [38]. Although our study aimed to expose the hidden curriculum, not to measure how common this hidden curriculum is, it is noteworthy that misconceptions were identified in all of the PowerPoints slides.

The hidden curriculum has mostly been studied within the “unofficial curriculum” [22], which refers to the learning that happens in the clinical environment through unscripted and interpersonal forms of teaching [20]. The presence of a hidden curriculum in PowerPoint slides, however, indicates that it might be more prevalent than anticipated. In fact, it is sometimes assumed that the content of what is being taught in the formal curriculum reflects the ideal “gold standard” of clinical medicine [38, 39], but our results question this assumption. The overarching presence of the hidden curriculum represents both a challenge and novel opportunities: improving attitudes around EBM has proved elusive [40], and addressing the hidden curriculum in formal classroom environment is worth exploring. Documents used to support teaching, such as PowerPoint slides, tutor guides, or self-learning modules, can be standardized and could be used to reinforce the use of EBM and SDM concepts to rationalize clinical decision making. Clinical decision making is not uniform and opinions regarding the “proper” management may differ among clinicians; however, these various courses of actions need to be grounded in the best available evidence, acknowledge limitations in the evidence, and patients values and preferences.

Role modeling has been shown to play an important role in transferring positive aspects of the hidden curriculum [41, 42] and EBM skills [43]. Our results suggest that PowerPoint slides should be considered as an additional medium to model EBM and SDM skills during day-to-day clinical didactic teaching. Furthermore, given that clinical teachers delivering formal didactic teaching may also be involved in informal clinical teaching, addressing the hidden curriculum in the formal classroom environment may also influence the use of EBM and SDM principles in informal teaching environments.

**Fig. 2:**
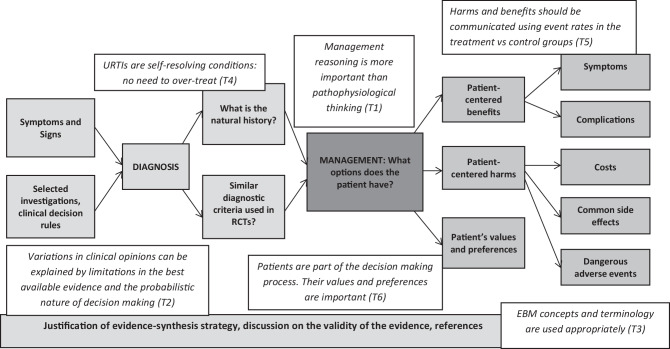
Concept mapping of a PowerPoint on the management of URTI that is consistent with SDM and EBM frameworks. Implicit messages are shown with the corresponding themes. This approach was rarely used and never in its entirety. *T1*: Emphasizing management reasoning over diagnostic binning and pathophysiological thinking, *T2*: Addressing uncertainties to explain variations in clinical care by using probabilistic approaches and by discussing the validity of the evidence, *T3*: Using appropriate EBM terminology, *T4*: Justifying choices of investigations and management plans by quantifying pre- and post-test probabilities and the benefits and harms of interventions, *T5*: Using event rates in the placebo and control groups to present harms and benefits in an unbiased way, *T6*: Explicitly mentioning the options that a patient has and the importance of his/her values and preferences

Our results provide insights to draw a new framework (Fig. 2) based on the IPDAS criteria and on the Sicily statement for evidence-based practice to model those skills. In this framework, authors of PowerPoint slides should (1) emphasize management reasoning over diagnostic binning and pathophysiological thinking, (2) address uncertainties to explain variations in clinical care by using probabilistic approaches and by discussing the validity of the evidence, (3) use appropriate EBM terminology, (4) justify choices of investigations and management plans by quantifying pre- and post-test probabilities and the benefits and harms of interventions, (5) use event rates in the placebo and control groups to present harms and benefits in an unbiased way, and (6) explicitly mention the options that a patient has and the importance of his/her values and preferences.

Our study has some limitations. To facilitate the analysis, we restricted the study to the diagnosis and management of URTIs. Our results would not necessarily be generalizable to other conditions. Moreover, it was assumed that the PowerPoint slides reflected the authors’ main messages. Recordings of the presentations were not available and it was not possible to ascertain whether oral comments differed substantially. However, students often use PowerPoint slides to prepare for examinations and the slides, in and of themselves, play an important role in their training. In addition, although most of the PowerPoints retrieved were from the USA, a minority were retrieved from countries that may not embrace EBM and SDM. Some of the PowerPoints were also published as long ago as 2001, when EBM and SDM were not as widely accepted as today. The misconceptions identified in our study might not have been characterized as such in those settings/era. However, it is important to note that the intent of the study was to reflect on whether different conceptualizations of EBM and SDM could be identified, and as such a diverse set of PowerPoints enriched the analysis. Finally, the same researcher performed the data collection and analysis. To address this limitation, reflexivity was maintained by ongoing discussions with researchers from the different fields of study.

## Conclusion

Formal curricular materials about URTIs, such as PowerPoint slides, can relay a hidden curriculum that may not align with SDM and EBM frameworks. Future research to develop best practices to address this hidden curriculum might represent a novel strategy to promote the uptake of EBM and SDM.

## Caption Electronic Supplementary Material

Supplemental Table 1: Characteristics of the PowerPoints
