# Supplementary material for: Evidence-based medicine, shared decision making and the hidden curriculum: a qualitative content analysis
Source: Perspect Med Educ. 2020 Apr 22;9(3):173–80. doi: 10.1007/s40037-020-00578-0 (PMC7283448; doi:10.1007/s40037-020-00578-0)
Supplement: Supplementary file 1 — Supplemental Table 1: Characteristics of the PowerPoints [file 40037_2020_578_MOESM1_ESM.docx]

**Supplemental Table 1:** **Characteristics of the PowerPoints**

| Publication year | Author: Qualifications, Affiliations | Nbr. slides |
| --- | --- | --- |
| PowerPoint P1  Publication year not reported, posted on slideshare.net in 2012 | Assistant professor  ENT Department HFH (USA) | 20 |
| PowerPoint P2  Publication year not reported, posted on slidshare.net in 2016 | MD, FACEP Professor of Military and Emergency Medicine  George Washington University (USA) | 73 |
| PowerPoint P3  Publication year not reported, Produced at least in 2011 given references | MD  Institute for Asthma and Allergy  George Washington University (USA) | 56 |
| PowerPoint P4  Publication year not reported, Produced at least in 2013 given references | MD  UT Health Science Center School of Medicine (USA) | 19 |
| PowerPoint P5  Publication year not reported | MD  Amiralam Hospital (Iran) | 48 |
| PowerPoint P6  Published in 2014 | Dr.  Medical Officer  ENT Department (Location NR) | 27 |
| PowerPoint P7  Publication year not reported | MD  Emory Family Medicine (USA) | 29 |
| PowerPoint P8  Published in 2001 | MD University of Texas, Department of Otolaryngology (USA) | 144 |
| PowerPoint P9  Publication year not reported  (pharyngitis) | Dr  Pediatric Infectious Diseases, Head infection control (Location NR) | 59 |
| PowerPoint P10  Publication year not reported | Dr  University of Pittsburgh (USA) | 31 |
| PowerPoint P11  Publication year not reported, Posted on slideshare.net in 2012 | Author NR  Department of Otorhinolaryngology  University of Davanagerere (India) | 19 |
| PowerPoint P12  Publication year not reported, Posted on slideshare.net in 2017 | Dr  Family Medicine Department (Location NR) | 41 |
| PowerPoint P13  Publication year not reported, Produced at least in 2015 given references | Qualifications NR  Consultant Medical Microbiologist  University of Cambridge School of Medicine (UK)  ** Explicit focus on antibiotic stewardship | 54 |
| PowerPoint P14  Published in 2008 | MD, MSPH  Professor of Medicine; epidemiology and biostatistics  University of California (USA)  ** Explicit focus on EBM | 44 |
| PowerPoint P15  Published in 2010 | Author NR  Published by the Annals of Internal Medicine  Ann Int Med. 152 (9): ITC5-1  ** Edited by peer-reviewed journal | 29 |
| PowerPoint P16  Publication year not reported, Produced at least in 2003 given references | MD  Published by American Academy of Family Physicians  ** CME provided by national organization | 81 |
| PowerPoint P17  Publication year not reported, Published on MedEdPortal in 2009 | Associate Professor of Medicine  Johns Hopkins University School of Medicine (USA)  Tomas P. Approach to cough: a team based leaning exercise. MedEdPORTAL. 2009;5:1312  ** Peer-reviewed, available on MedEdPortal | 68 |
| PowerPoint P18  DECISION + workshop  Results were Published in 2012 in CMAJ | France Legare, Michel Labrecque, Michel Cauchon  Professors of Family Medicine  Université Laval (Canada)  ** explicit focus on SDM | 75 |

^Abbreviations. NR: not reported^
